# PinX1 suppresses cancer progression by inhibiting telomerase activity in cervical squamous cell carcinoma and endocervical adenocarcinoma

**DOI:** 10.1016/j.gendis.2024.101319

**Published:** 2024-05-07

**Authors:** Yue Weng, Xiangyu Yan, Biying Chen, Zhouliang Bian, Yunhui Ge, Hong Lu, Shufang He, Jian Wu, Yong Chen, Ming Lei, Yanjie Zhang

**Affiliations:** a Shanghai Institute of Precision Medicine, Ninth People's Hospital, Shanghai Jiao Tong University School of Medicine, Shanghai 200125, China; b State Key Laboratory of Oncogenes and Related Genes, Shanghai Jiao Tong University School of Medicine, Shanghai 200025, China; c Department of Oncology, Ninth People's Hospital, Shanghai Jiao Tong University School of Medicine, Shanghai 201900, China; d State Key Laboratory of Molecular Biology, Shanghai Institute of Biochemistry and Cell Biology, Center for Excellence in Molecular Cell Science, Chinese Academy of Sciences, Shanghai 200031, China; e University of Chinese Academy of Sciences, Beijing 100049, China; f School of Life Science and Technology, ShanghaiTech University, Shanghai 201210, China

Telomerase plays an essential role in the immortalization and stemness of cancer cells. PIN2/TRF1-interacting telomerase inhibitor 1 (PinX1) functions as a telomerase inhibitor and tumor suppressor.^1^ However, the underlying mechanism is still not clear. Here, we report the molecular basis of the tumor suppression function of PinX1. We determined the crystal structure of the TRFH (TRF homology) domain of TRF1 in complex with a short TRF1-binding motif of PinX1 and revealed that PinX1 bound to TRF1 via the F-X-L-X-P motif, providing a structural basis of how PinX1 is recruited to the telomeric region by TRF1. We demonstrated that PinX1 is directly associated with and inhibits telomerase activity by TID (telomerase inhibitory domain) with crucial lysine residues clustered in PinX1_292-301_. In cervical squamous cell carcinoma and endocervical adenocarcinoma (CESC), which is featured by short telomeres and high telomerase activity, PinX1-TID efficiently inhibits cancer stemness traits by primarily targeting telomerase activity. These findings provide valuable insights for developing strategies to treat cancers with short telomeres and advancing telomerase inhibitor therapeutics.

Systematic analysis of telomere length and telomerase activity using multidimensional datasets revealed that CESC markedly displayed a prominent short telomere and high telomerase activity (Fig. S1A and Table S1) among most cancer types, suggestive of a dramatic telomerase dependency, thus conferring an optimal model to explore the telomerase regulatory mechanism (Fig. S1A). Homozygous and heterozygous deletion of PinX1 occurred in approximately one-third of CESC patients (94 out of 278) and was associated with low PinX1 mRNA level and high telomerase activity (Fig. S1B, C). Furthermore, PinX1 deletion significantly correlated with increased cancer stemness (Fig. S1D, E) and poor prognosis (Fig. S1F, G) in CESC and even pan-cancer analysis (Fig. S1H–L). In three CESC cell lines (Figs. S2A–C), PinX1 deletion by shRNA led to a significant increase in cell viability (Fig. 1A; Fig. S2D, E), colony formation (Fig. 1B; Fig. S2F, G), migration (Figs. S2H–J), invasion (Fig. S2K–M), and cisplatin resistance (Fig. S2N–P). These findings highlight that PinX1 dysfunction might be necessary to allow tumor progression towards more malignant states, contributed at least partially by the subsequent activation of telomerase.

**Figure 1:**
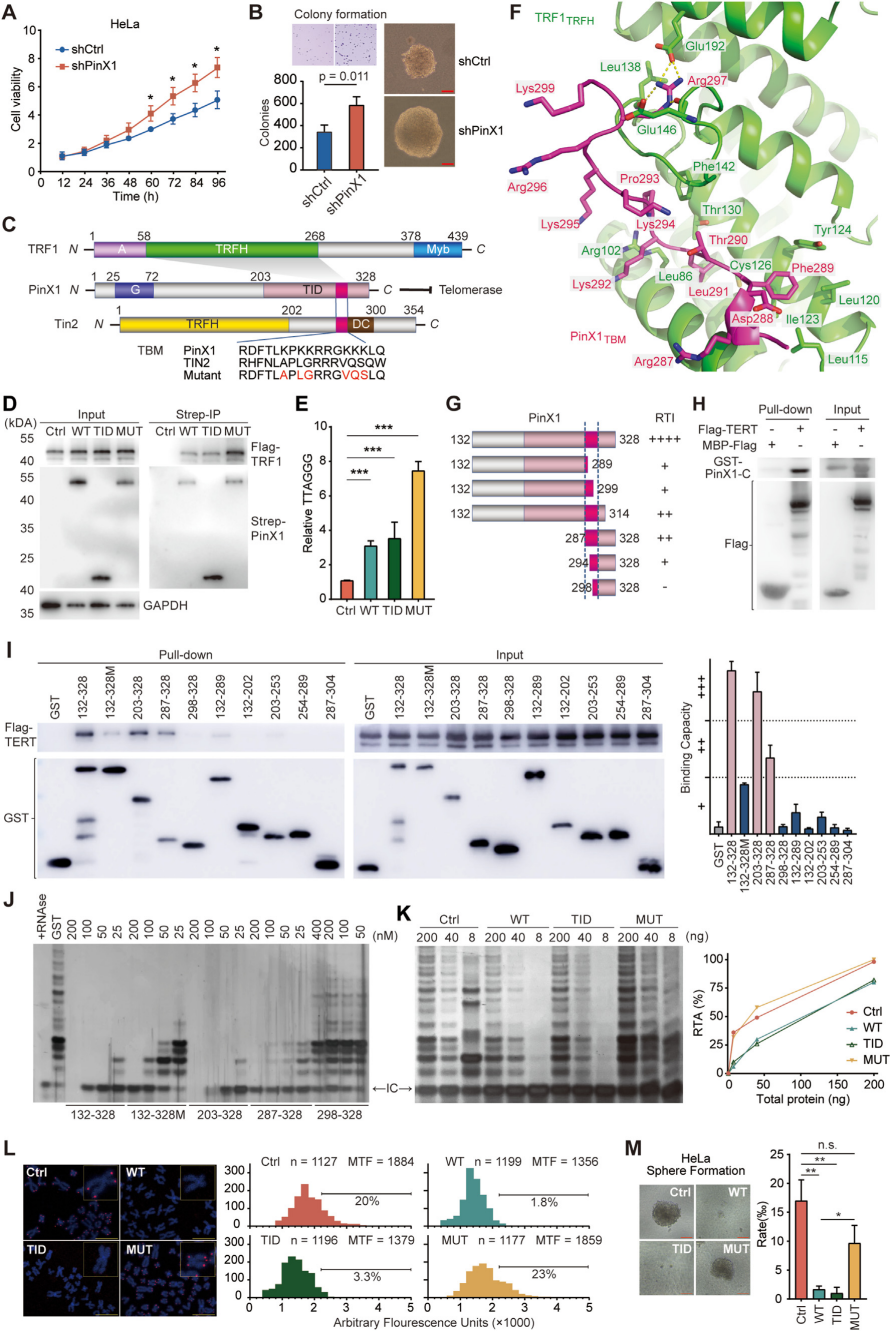
PinX1 directly binds to telomerase and inhibits its activity to suppress cancer progression in cervical squamous cell carcinoma and endocervical adenocarcinoma. **(A)** Growth curves of shCtrl and shPinX1 HeLa cells were recorded using RTCA (real-time cellular analysis). The student's *t*-tests were used to analyze the differences at 60, 72, 84, and 96 h respectively, ∗*P* < 0.05. **(B)** Colony formation of shCtrl and shPinX1 HeLa cells in soft agar. Representative images for crystal violet staining and colonies were shown. Scale bar, 100 μm. The *P*-value was calculated with the student's *t*-test. **(C)** Organization of the TRF1 and PinX1 polypeptide chains and the mutant of PinX1. A (acidic) region of TRF1 was colored lilac. TRFH (TRF homology) domain of TRF1 and TIN2 were colored green and yellow respectively. Telomeric DNA binding motif Myb of TRF1 was colored blue. G (Gly-rich) region of PinX1 was colored bluish violet. The TID (telomerase inhibitory domain) of PinX1 was colored pink. TBM (TRFH-binding motif) was colored red. DC (dyskeratosis congenita hotspot) was colored brown. The shaded areas indicated the interactions between PinX1 and TRF1. **(D)** Co-immunoprecipitation assays of Flag-TRF1 with Strep-PinX1 using HeLa cell lysates. WT, 2 × Strep-PinX1_2-328_; TID, 2 × Strep-PinX1_203-328_; MUT, 2 × Strep-PinX1_2-328 (MUT292-301)_. **(E)** Chromatin immunoprecipitation-quantitative PCR analysis for telomere repeats (TTAGGG)n–PinX1 binding in HeLa cells. The *t*-tests adjusted by false discovery rate were used for statistical analysis; ∗∗∗*P* < 0.001. **(F)** Superposition of the TRF1_TRFH_-PinX1_TBM_ complex on the unliganded structure of TRF1_TRFH_. **(G)** Alignment of GST-PinX1 C-terminal truncations showing the crucial motif, colored red. **(H)** Western blot verification for pull-down assays of GST-PinX1-C (132–328) captured by MBP-3 × Flag and 3 × Flag-TERT. **(I)** Pull-down assays of 3 × Flag-TERT captured by different constructions of GST-PinX1 C-terminal. 132–328M indicates GST-PinX1_132-328 (MUT292-301)_. Quantification was presented in the right panel. **(J)** The impact of GST-PinX1 C-terminal truncations on purified telomerase was analyzed by TRAP assays. IC, internal control. **(K)** TRAP assays and statistics of RTA (relative telomerase activity) for ectopically expressed PinX1 constructions using quantified HeLa cell lysates. **(L)** Quantitative fluorescence *in situ* hybridization analysis and statistics of telomere length in HeLa cells expressing different PinX1 constructions. Scale bar, 10 μm. MTF, mean telomere fluorescence. **(M)** Representative images and statistics of sphere formation of HeLa cells expressing different PinX1 constructions. Quantification was presented in the right panel. Scale bar, 100 μm. The *t*-tests adjusted by false discovery rate were used for statistical analysis; n.s., *P* > 0.05; ∗*P* < 0.05, ∗∗*P* < 0.01, ∗∗∗*P* < 0.001.

We have reported the crystal structure of the telomeric TIN2-TRF1 complex previously, revealing that the short TBM (TRFH-binding motif) of TIN2 directly binds to the TRFH of TRF1.^2^ Notably, an 11-residue fragment of PinX1 (R287-D-F-T-L-K-P-K-K-R-R297), referred to as PinX1_TBM_, closely resembles TIN2_TBM_, indicating that it may bind to TRF1_TRFH_ in the same fashion as TIN2_TBM_ does (Fig. 1C). To test this idea, we conducted co-immunoprecipitation and chromatin immunoprecipitation-quantitative PCR assays and demonstrated that PinX1 indeed interacted with TRF1 and telomeric DNAs in cells (Fig. 1D, E). To provide direct and independent evidence of the PinX1-TRF1 interaction, we purified a high-quality TRF1_TRFH_-PinX1_TBM_ complex and determined its crystal structure at a resolution of 2.7 Å (Fig. S3A and Table S2). The structure showed that TRF1_TRFH_ formed homodimers, with each TRF1_TRFH_ interacting with one PinX1_TBM_ peptide (Fig. S3B). The electron density map demonstrates that the PinX1_TBM_ peptide assumes a well-defined conformation (Fig. S3C). Similar to the TIN2_TBM_-TRF1_TRFH_ interface, hydrophobic contacts are essential for the interaction between PinX1_TBM_ and TRF1_TRFH_ (Fig. 1F).^2^ L291 of PinX1 was deeply buried in a pocket formed by L86, R102, C126, and T130 of TRF1 (Fig. 1F), supporting previous findings that the L291E mutation in PinX1 disrupts its ability to be bound and recruited by TRF1.^2^^,^^3^ The aromatic side-chain of F289 of PinX1 packed against a hydrophobic surface of TRF1 and the proline ring of PinX1 P293 stacked with TRF1 F142 (Fig. 1F). Notably, except for R297 of PinX1 that is engaged in electrostatic interactions with E146 and E192 of TRF1, all other basic residues in PinX1_TBM_ (K292, K294, K295, R296, and K299) extended outward from the interface and did not contribute to the interaction with TRF1 (Fig. 1F).

To examine the motif of PinX1 that inhibits telomerase, we constructed different GST-PinX1 C-terminal truncations in the frame of 132–328 aa based on the previous report,^4^ and performed TRAP (telomeric repeat amplification protocol) assays to evaluate their RTI (relative telomerase inhibition). The RTIs of different truncations (Figs. S4A–C) suggest that residues 287–328 of PinX1 are essential for the telomerase inhibitory function, with crucial amino acids located within PinX1_287-299_ (Fig. 1G). We also conducted a series of PinX1_287-301_ mutants and found that mutations of basic amino acids destroyed RTI more substantially (Fig. S4D, E). PinX1_MUT_ (A292-P-L-G-R-R-G-V-Q-S301) almost completely abolished PinX1 RTI in the TRAP assay (Fig. S4F, G), although the TRFH binding capability was unaffected (Fig. 1D), suggesting that lysine residues in PinX1_292-301_ are essential for the telomerase inhibitory activity of PinX1. Pull-down assays of purified telomerase (Fig. S5A) and PinX1 showed direct interaction between them (Fig. 1H; Figs. S5B–D). Moreover, the binding ability of various truncations or the mutant is also different in accordance with those RTI data (Fig. 1I, J; Fig. S5E, F). Based on these observations, we conclude that PinX1_203-328_, hereafter referred to as TID, confers a robust capacity to directly bind to and inhibit telomerase with key lysine residues in 292–301 (Fig. S5F).

Consistent with the *in vitro* data, HeLa cells stably expressing PinX1_wild-type (WT)_ or PinX1_TID_ exhibited dramatically decreased levels of telomerase activity relative to the control, while PinX1_MUT_ did not show a similar effect (Fig. 1K). In alignment with diminished telomerase activity, PinX1_WT_ and PinX1_TID_ expressing cells exhibited drastically shortened telomere revealed by quantitative fluorescence *in situ* hybridization assay, while the telomere length in PinX1_MUT_ maintained stable (Fig. 1L; Figs. S6A–C). Then we investigated the impact of PinX1 on stemness-associated phenotypes and found that telomerase activity was closely correlated with cancer stemness in CESC and even in pan-cancer (Fig. S6D, E). In three CESC cell lines, expression of PinX1_WT_ or PinX1_TID_ but not PinX1_MUT_ substantially suppressed sphere formation (Fig. 1M; Fig. S6F, G). Furthermore, PinX1_WT_ or PinX1_TID_ expression dramatically suppressed stemness traits including migration, invasion (Figs. S7A–C), cisplatin resistance (Figs. S7D–F), and cell growth (Figs. S7G–I), while PinX1_MUT_ failed to restrain this aggressiveness. Collectively, PinX1 shortens telomeres and suppresses cancer stemness in CESC through the inhibition of telomerase activity, and TID with lysine residues clustered in PinX1_292-301_ are crucial for this suppressive effect.

Telomere maintenance via telomerase reactivation is a nearly universal hallmark of cancer cells. Telomerase inhibitors have historically been heralded as promising anti-cancer agents. As a natural suppressor of telomerase, PinX1 represents a potential strategy for curtailing cancer progression by targeting telomerase.^1^^,^^5^ This study reported the crystal structure of the TRF1_TRFH_-PinX1_TBM_ complex and confirmed that PinX1 was recruited to the telomere by TRF1 predominantly via the binding to the F-X-L-X-P motif, highlighting its tumor-suppressive impact on CESC (Fig. S8). This discovery offers inspiration for the treatment of short telomere cancers and has implications for the development of anti-telomerase medicine by targeting the telomeric protein docking site.

## Author contributions

Yue Weng and Xiangyu Yan contributed equally to this work. Yong Chen, Ming Lei, and Yanjie Zhang designed/conceived the study. Yue Weng, Xiangyu Yan, Biying Chen, Zhouliang Bian, Yunhui Ge, Hong Lu, Shufang He, and Jian Wu conducted the experiments. Ming Lei and Yanjie Zhang wrote the manuscript. All authors read and approved the final manuscript.

## Funding

This work was supported by grants from the 10.13039/501100001809National Natural Science Foundation of China (No. 31930063 to Ming Lei, 82072638 to Yanjie Zhang, 32100582 to Yunhui Ge), Innovative Research Team of High-level Local University in Shanghai, China (No. SHSMU-ZLCX20211700 to Ming Lei), and Shanghai Municipal Health Commission and Collaborative Innovation Cluster Project (China) (No. 2019CXJQ01 to Ming Lei).

## Conflict of interests

The authors declare that there is no competing interests.

## Figures and Tables

**figs1:**
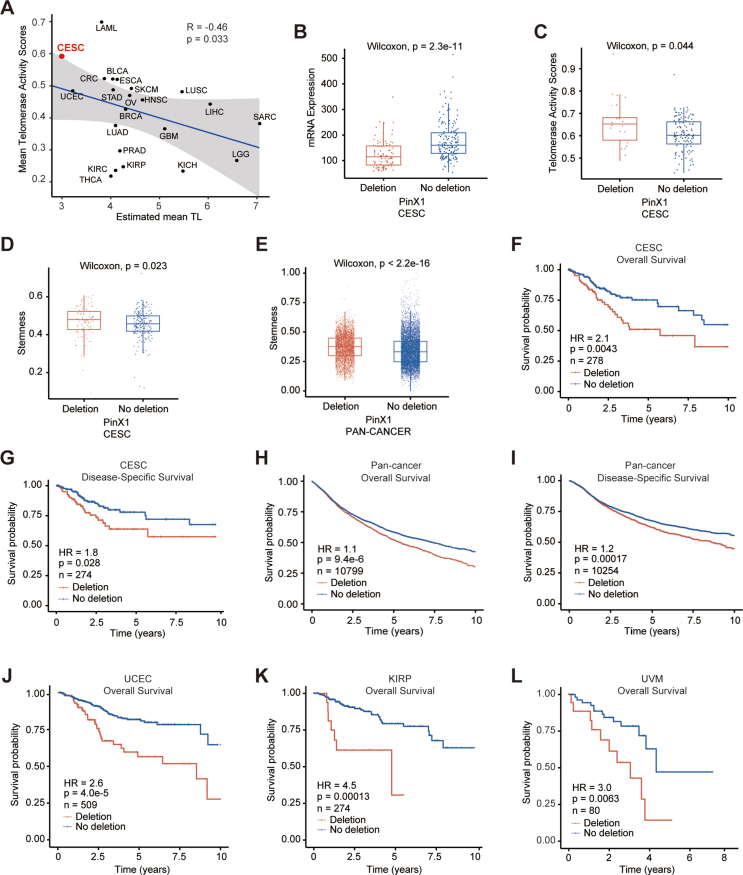
PinX1 deletion correlates with poor prognosis in TCGA dataset. **(A)** Scatterplot of multiple TCGA projects ranking by telomere length and telomerase activity. Tumor types with fewer than 10 samples were excluded. CESC was highlighted in red. The R and p value were calculated by Spearman rank correlation test. **(B)** Comparison of PinX1 mRNA levels in different PinX1 gene states in CESC. The p value was calculated with the Wilcoxon rank sum test. **(C)** Comparison of telomerase activity between different PinX1 gene states in CESC. The p value was calculated with the Wilcoxon rank sum test. **(D, E)** Comparison of cancer stemness between different PinX1 gene states in CESC and pan-cancer. The p values were calculated with the Wilcoxon rank sum tests. **(F, G)** Kaplan-Meier overall survival and disease-specific survival curves for patients with or without PinX1 deletion in CESC. HRs (Hazard ratios) were estimated with the Cox models, and p values were calculated with the log-rank tests. **(H, I)** Kaplan-Meier overall survival and disease-specific survival curves for patients with or without PinX1 deletion in pan-cancer. HRs were estimated with the Cox models, and p values were calculated with the log-rank tests. **(J-L)** Overall survival of patients with or without PinX1 deletions in UCEC (Uterine Corpus Endometrial Carcinoma), KIRP (Kidney renal papillary cell carcinoma) and UVM (Uveal Melanoma). HRs were estimated with the Cox models, and p values were estimated with the log-rank tests.

**figs2:**
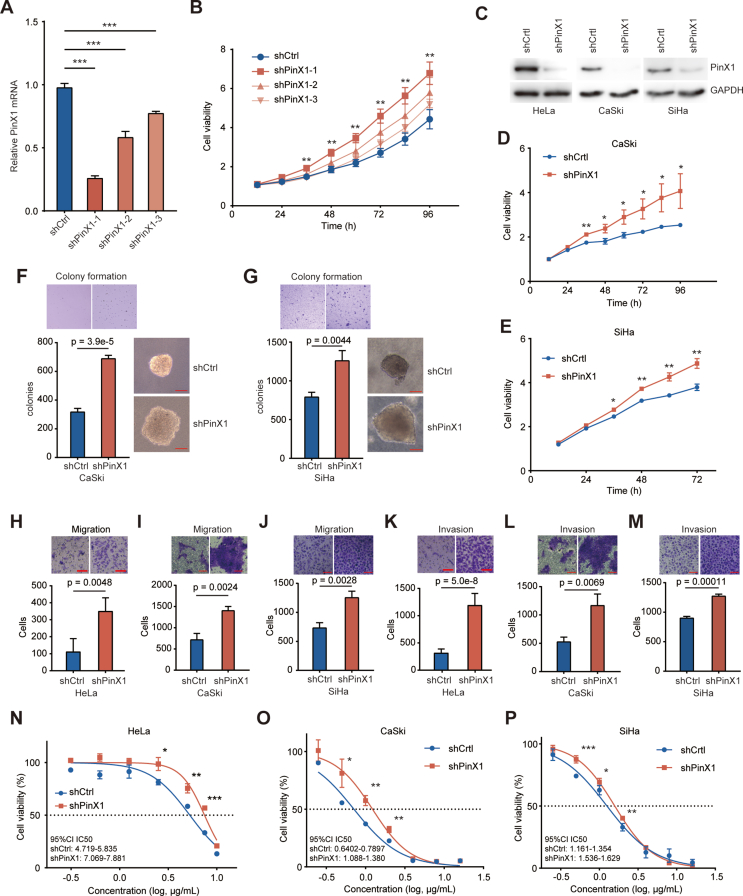
PinX1 deletion correlates with cancer aggressiveness. **(A)** shRNA screening for PinX1 knockdown in HeLa cells. Knockdown efficiency was examined by real-time qPCR. The t-tests adjusted by FDR were used for statistical analysis, ∗∗∗p<0.001. **(B)** Growth curves of HeLa cells stably transfected with shCtrl, shPinX1-1, shPinX1-2 or shPinX1-3 were recorded using the RTCA. The t-tests adjusted by FDR were used to analyze the differences between shCtrl and shPinX1-1 at 36, 48, 60, 72, 84, 96 hours respectively, ∗∗p<0.01. shPinX1-1 was selected for further experiments, termed as shPinX1 in this study. **(C)** Knockdown of PinX1 in HeLa, CaSki and SiHa cells was confirmed by Western blot. **(D, E)** Growth curves of shCtrl and shPinX1 CaSki/SiHa cells were recorded using the RTCA. The p values were calculated with the Student’s t-tests. **(F, G)** Quantification of the colony formation of shCtrl and shPinX1 CaSki/SiHa cells in soft agar. Representative images for crystal violet staining and colonies were shown. Scale bar, 100 μM. The p values were calculated with the Student’s t-tests. **(H-M)** Quantification of the migration and invasion of shCtrl and shPinX1 HeLa/CaSki/SiHa cells. Representative crystal violet staining images were shown. Scale bar, 100 μM. The p values were calculated with the Student’s t-tests. **(N-P)** Dose-response curves of shCtrl and shPinX1 HeLa/CaSki/SiHa cells. The IC50s (50% inhibitory concentrations) of cisplatin we labeled on the graph. The Student’s t-tests were used to analyze the differences at the certain concentrations respectively, ∗p<0.05, ∗∗p<0.01, ∗∗∗p<0.001. CI, confidence interval.

**figs3:**
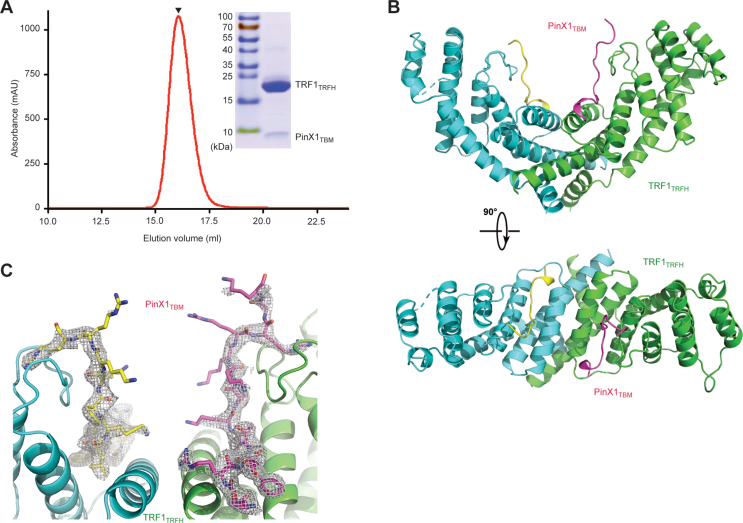
Structure of the TRF1_TRFH_-PinX1_TBM_ complex. **(A)** The gel filtration chromatography (Superdex 200) and SDS-PAGE gel verification of the purified TRF1_TRFH_-PinX1_TBM_ complex. **(B)** Overall structure of the dimeric TRF1_TRFH_-PinX1_TBM_ complex. **(C)** Electron density map of PinX1_TBM_.

**figs4:**
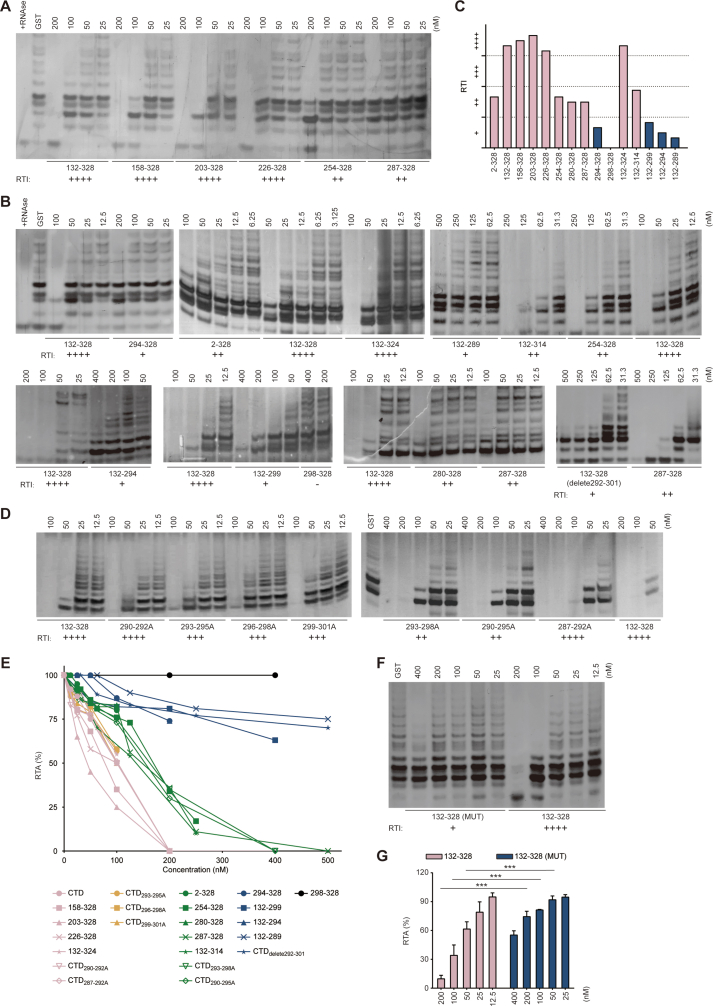
Telomerase inhibition by GST-PinX1 C-terminal truncations and mutants. **(A)** The impact of PinX1 truncations on telomerase activity was analyzed by TRAP assays. - ∼ ++++ indicated increasing gradient of RTI (relative telomerase inhibition) (the same as in panel B-D, F, G). **(B)** TRAP assays of RTI for different GST-PinX1 C-terminal truncations. **(C)** Quantification of panel A, B. **(D)** TRAP assays of RTI for different GST-PinX1_132-328_ mutants. **(E)** Quantification of TRAP signals from different GST-PinX1 C-terminal truncations. RTA, relative telomerase activity. CTD (C-terminal domain), GST-PinX1_132-328_. **(F)** The impact of GST-PinX1_132-328_ and GST- PinX1_132-328 (MUT)_ on telomerase activity was analyzed by TRAP assays. **(G)** Quantification of panel F. The experiment was conducted three times and statistically analyzed by the t-tests adjusted by FDR, ∗∗∗p<0.001. RTA, relative telomerase activity.

**figs5:**
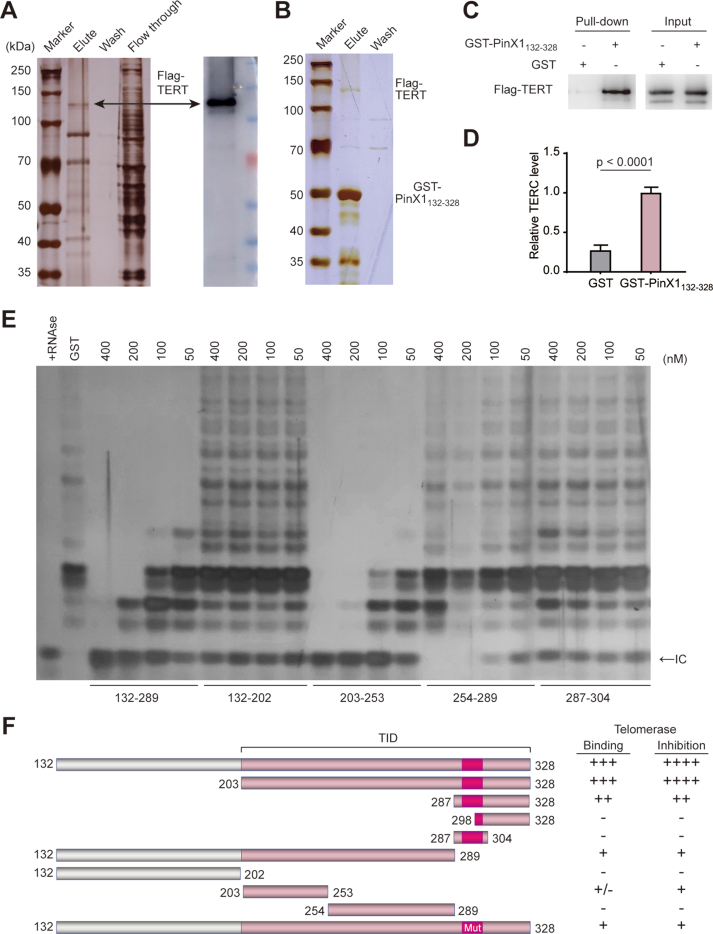
TID-mediated direct binding and inhibition of PinX1 on purified telomerase. **(A)** Human telomerase was purified, and verified by SDS-PAGE silver staining and Western blot. **(B)** SDS-PAGE silver staining for pull-down assays of 3×Flag-TERT captured by GST-PinX1_132-328_. **(C)** Western blot verification for pull-down assays of 3×Flag-TERT captured by GST and GST-PinX1_132-328_. **(D)** RT-qPCR of TERC (Telomerase RNA component) pulled-down by GST and GST-PinX1_132-328_. The p value was calculated with the Student’s t-test. **(E)** The impact of GST-PinX1 C-terminal truncations on purified telomerase was analyzed by TRAP assays. IC, internal control. **(F)** Capacity of variant PinX1 truncations for binding and inhibiting telomerase. The increasing binding capacity was indicated by - ∼ +++.­ The increasing gradient of RTI was indicated by - ∼ ++++.

**figs6:**
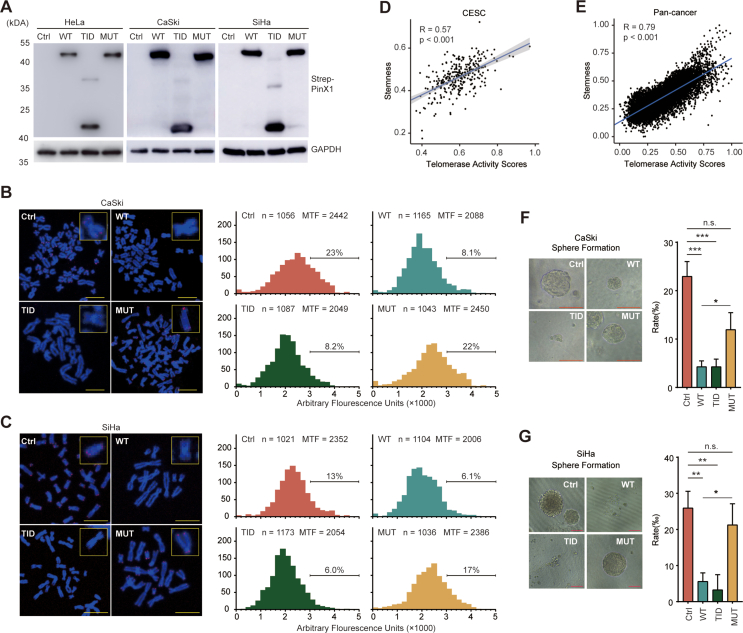
PinX1 suppresses cancer stemness through the inhibition of telomerase activity. **(A)** 2×Strep tagged-PinX1_2-328_ (WT), -PinX1_203-328_ (TID), and -PinX1_2-328 (MUT292-301)_ (MUT) were expressed in HeLa, CaSki and SiHa cells, and confirmed by Western blot. **(B, C)** Q-FISH analysis and statistics of telomere length in CaSki and SiHa cells expressing different PinX1 constructions. Scale bar, 10 μM. MTF, mean telomere fluorescence. **(D, E)** Correlation analysis of telomerase activity and cancer stemness in CESC and pan-cancer. The R and p value were calculated by Spearman rank correlation test. **(F, G)** Representative images and statistics of sphere formation of CaSki and SiHa cells expressing different PinX1 constructions. Scale bar, 100 μM.

**figs7:**
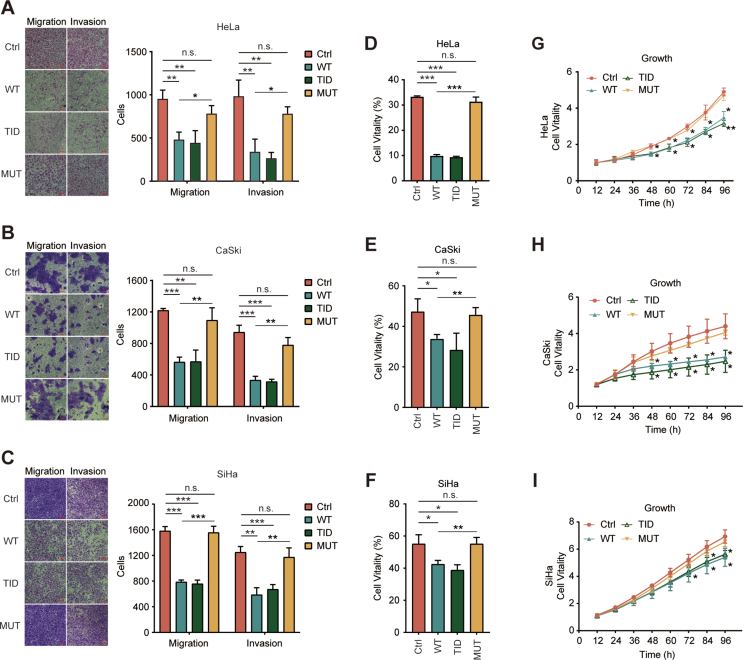
Suppression of PinX1 on Cancer aggressiveness. **(A-C)** Crystal violet staining and statistics of migration and invasion in HeLa, CaSki and SiHa cells expressing different PinX1 constructions. Scale bar, 100 μM. **(D-F)** HeLa, CaSki and SiHa cells expressing different PinX1 constructions were treated with cisplatin (HeLa: 7.5 μg/mL, CaSki and SiHa: 1 μg/mL) for 3 days. Cell viability was measured by SRB assay. **(G-I)** Growth curves of HeLa, CaSki and SiHa cells expressing different PinX1 constructions were recorded using the RTCA. All statistics were performed using the t-tests adjusted by FDR, n.s. p>0.05, ∗p<0.05, ∗∗p<0.01, ∗∗∗p<0.001.

**figs8:**
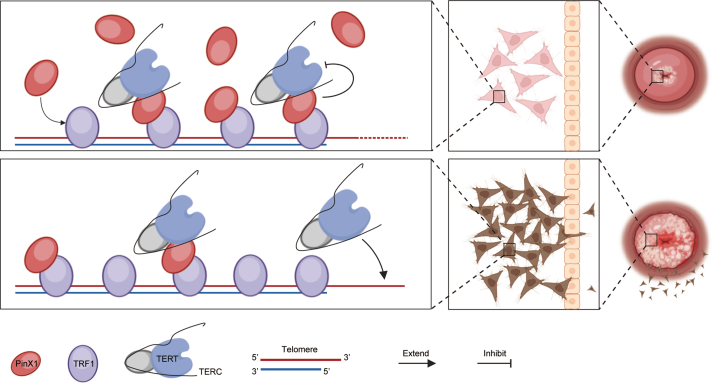
Schematic depicting a tumor suppressive effect of PinX1 in CESC by telomerase inhibitionWe propose that PinX1 is recruited to telomeres by TRF1, and subsequently binds to telomerase and inhibits its activity, resulting in a tumor-suppressive effect on CESC (top). In cancers, PinX1 deletion unleashes telomerase activity to elongate telomeres, and contributes to tumor progression (bottom).
