# Supplementary material for: PinX1 suppresses cancer progression by inhibiting telomerase activity in cervical squamous cell carcinoma and endocervical adenocarcinoma
Source: Genes Dis. 2024 May 7;12(2):101319. doi: 10.1016/j.gendis.2024.101319 (PMC11615109; doi:10.1016/j.gendis.2024.101319)
Supplement: Multimedia component 1 [file mmc1.docx]

## Materials and Methods

### Analysis of TCGA (The Cancer Genome Atlas) data

TPM (Transcripts Per Million) normalized transcriptional data were obtained from UCSC Xena data hub, along with corresponding information on PinX1 gene deletion status, patient overall survival and disease-specific survival from the TCGA Pan-Cancer Atlas study in cBioPortal. Patients were stratified into two groups based on PinX1 deletion status: deletion or non-deletion. Kaplan-Meier survival curves were created with survfit from R package survival and plotted with ggsurvplot from R package survminer. The TL (telomere length), mRNA stemness index and telomerase activity scores of the corresponding cancer samples were obtained from references^1,2^. Wilcoxon rank sum tests were performed to compare telomerase activity and stemness respectively between the PinX1 deletion and non-deletion groups. A correlation analysis was conducted to examine the relationship between TL and telomerase activity in 22 different types of cancer. Additionally, the correlation between stemness and telomerase activity was analyzed in CESC or pan-cancer samples.

### Cell lines

293T (ATCC, Cat. CRL-3216), HeLa (ATCC, Cat. CCL-2), CaSki (ATCC, Cat. CRL-1550) and SiHa (ATCC, Cat. HTB-35) cells were maintained in DMEM High glucose with L-glutamine, sodium pyruvate (Hyclone, Cat. SH30243.01) or RPMI 1640 medium modified with L-glutamine (Hyclone, Cat. SH30809.01) supplemented with 10% fetal bovine serum (FBS, Gibco, Cat. 10099141C) and 1% penicillin-streptomycin (Hyclone, Cat. SV30010) at 37 ℃ in 5% CO_2_. Tc6-3 cells (the Expi293F cells (Gibco, Cat. A14527) stably expressing TCAB1)^3^ were maintained in Union-293 medium (Union-Biotech, Cat. UP0050) at 37 ℃ in 5% CO_2_.

### Construction of the recombinant lentiviral vectors and stable cell lines

The pLKO-shCtrl, pLKO-shPinX1-1, pLKO-shPinX1-2 and pLKO-shPinX1-3 lentivirus vectors were constructed by ligating oligonucleotide linkers (Table S3, No. 1-8) and linearized pLKO with T4 DNA ligase (Thermo Scientific, Cat. EL0012). The reconstructed plasmids were transfected into 293T cells along with two packaging plasmids pCMV-dR8.2 dvpr (Addgene, Cat. 8455) and pCMV-VSV-G (Addgene, Cat. 8454) to generate recombinant lentivirus particles using Lipofectamine 3000 Reagent (Thermo Scientific, Cat. L3000015). The supernatants were harvested after 48-72 h, and subjected to filtering using 0.45 μm sterile filter. Based on the results of RT-qPCR analysis, shPinX1-1 was selected for subsequent experiments, termed as shPinX1.

Additionally, the PCR products of the PinX1 gene and its mutants (amplified by primers, Table S3, No. 9-11, 85, 86) were inserted into a modified pLVX-IRES-Puro lentivirus vector to construct 2×Strep tagged PinX1 WT or mutations over-expressed plasmids. And then the plasmids were packaged as described above. The cells were infected with the lentivirus particles, and puromycin (Gibco, Cat. A11138-03) was added at a final concentration of 2 μg/mL for 2-3 days to select stable cell lines.

### RTCA (Real-time Cell Analyzer Assay)

A real-time cell analyzer xCELLigence (ACEA Biosciences) was used to monitor cell growth according to the manufacturer’s instruction. Briefly, HeLa (3000 cells/well), SiHa (1500 cells/well) and CaSki (3000 cells/well) were seeded in E-plate 96 and real-time electric resistance values were measured for 72-96 h. Cell growth curves were presented as real-time measurement of unitless cell indices using the formula: cell index = (R_Tn_- R_T0_) /15 *Ω* (R_Tn_: the resistance measured at a time point Tn, R_T0_: the background resistance measured at time point T0).

### Cisplatin resistance analysis

IC50 (50% inhibitory concentration) of cisplatin was assessed by SRB (Sulforhodamine B) assay. The cells (HeLa, 5000 cells/well; CaSki, 5000 cells/well; SiHa, 2500 cells/well) were seeded in 96-well plates and incubated for 72 h after the addition of varying concentrations of cisplatin (Sigma-Aldrich, Cat. 232120). Subsequently, the cells were fixed with 25% TCA (Sigma-Aldrich, Cat. T0699) at 4 ℃ for 1 h, followed by washing and drying. The cells were then stained with 0.4% SRB (Sigma-Aldrich, Cat. S9012) solution at room temperature for 30 min. After washing with 1% acetic acid and drying, 10 mM Tris (pH 10.5) was added and incubated at room temperature for 30 min. The optical density at 520 nm was then measured to determine cell viability: cell viability = (A_X_-A_Blank_)/(A_0_-A_Blank_) (A_X_: the absorbance measured at X concentration of cisplatin; A_Blank_: the background absorbance (cell-free); A_0_: the absorbance measured at none of cisplatin). The cisplatin resistance curve was plotted using GraphPad software (8.0.2).

### Analysis of cell migration, invasion

For the migration assay, a volume of 200 μL serum-free medium containing a specific number of cells (5×10^4^ cells/well) was seeded in the upper chamber of Transwell pore plate (Corning, Cat. CLS3422). 600 μL complete medium was added to the lower chamber. Following a 48-h incubation, cells were fixed and stained before being subjected to microscopic analysis. For the invasion assay, a serum-free medium mixed with MatriGel (Corning, Cat. 356234) at a ratio of 9:1 was pre-added to the upper chamber. Then 10^5^ cells were seeded in the upper chamber. The subsequent steps were identical to the migration procedure described above.

### Anchorage-independent growth assay

Soft agar colony formation assay was performed as described previously^4^. Underlying gels containing 0.6% low-melting agarose, 10% FBS, 1% penicillin-streptomycin, 1×DMEM High glucose (Genom, Cat. 12802-2) or RPMI 1640 medium (Genom, Cat. 31802-2) was added in a 6-well plate. Following overnight coagulation, a mixture of cells (2000 cells/well) and upper gel solution (0.35% low-melting agarose, 10% FBS, 1% penicillin-streptomycin, 1×DMEM High glucose or RPMI 1640 medium) was prepared and coated onto the lower gel. After solidification, the complete medium was added for cultivation. After 4-8 weeks, the colonies in soft agar were observed under an inverted microscope, stained with 0.01% crystal violet and counted.

### Sphere Formation Assay

Sphere formation assay was performed as described previously^5^. The cells were suspended in a cancer stem cell medium consisting of serum-free DMEM/F12 medium (Hyclone, Cat. SH30023.01) with 1% penicillin-streptomycin, 20 ng/mL epidermal growth factor (R&D, Cat. 236-EG), 10 ng/mL basic fibroblast growth factor (R&D, Cat. 3718-FB), and 2% B27 (Gibco, Cat. A3582801). The mixture was combined with MatriGel at a 1:1 ratio and spread uniformly in 96-well plates with 500 cells per well. Once the MatriGel solidified, the cancer stem cell medium was added and changed every 3 days. After 2 weeks, the cell spheres were imaged by inverted microscope, and the number of cell spheres was counted.

### Protein Expression and Purification

The PCR products of human TRF1_TRFH_ (residues 65-269, amplified by primers, Table S3, No. 12, 13) was cloned into a modified pET28a vector with a SUMO protein fused at the N-terminus after the 6×His tag. PinX1_TBM_ (residues 285-304, annealed by primers, Table S3, No. 14, 15) was cloned into a modified pGEX-6P1 vector with a GST tag fused at the N-terminus. Additional versions of PinX1 (amplified by primers, Table S3, No. 16-20, 37-86) were also cloned into the pGEX-6P1 vectors. The proteins were co-expressed or expressed alone in E. *coli* BL21(DE3). After induction for 16 h with 0.1 mM IPTG (Isopropyl-beta-D-thiogalactopyranoside) at 25 ℃, the cells were harvested by centrifugation and the pellets were resuspended in lysis buffer containing 50 mM Tris-HCl pH 8.0, 50 mM NaH_2_PO_4_, 400 mM NaCl, 10% glycerol, 1 mM PMSF (phenyl methane sulfonyl fluoride), 0.1 mg/ml lysozyme, 2 mM β-mercaptoethanol, and Protease Inhibitor Cocktail (MCE, Cat. HY-K0010). The cells were then lysed by sonication and the cell debris was removed by ultracentrifugation.

For purification of TRF1_TRFH_-PinX1_TBM_ complex, the supernatant was mixed with Ni-NTA agarose beads (Qiagen, Cat. 30210) with 3 mM imidazole and rocked for 4 h at 4 ℃ before elution with 250 mM imidazole. Then SUMO proteases (Beyotime, Cat. P2312S) and PreScission proteases (Beyotime, Cat. P2302) were added to remove the His-SUMO and GST tags at 4 ℃ overnight. The proteins were further purified by Mono-Q (GE Healthcare) and then rebound with glutathione agarose (Thermo Scientific, Cat. 16101) to remove the trace GST tag. After a final step of gel-filtration chromatography on a Superdex 200 column (GE Healthcare) equilibrated with 25 mM Tris-HCl pH 8.0, 150 mM NaCl and 5 mM dithiothreitol, the purified proteins were concentrated to 25 mg/mL and stored at -80 ℃.

For PinX1 and its mutants, the supernatant was mixed with GST beads (GE Healthcare, Cat. 17075601) and incubated for 4 h at 4 ℃. The bound protein was washed with buffer P (25 mM Tris-HCl pH 8.0, 200 mM NaCl) before elution with 15 mM L-Glutathione Reduced. The preliminary purified fusion proteins were further purified using Hitrap Heparin HP 5 mL (GE Healthcare) at a linear gradient of increasing salt concentration (200-800 mM NaCl), and finally equilibrated by Superdex 200 column with buffer P. The purified PinX1 protein and its mutants were stored at -80 ℃ for further use.

### Crystallization, Data Collection and Structure Determination

Crystals were grown by hanging-drop-vapor-diffusion at 16 ℃. The precipitant/well solution contains 4% PEG2000 MME, 50 mM HEPES pH 7.6, 3% 1.6-hexanediol and 40 mM NaCl. Crystals were gradually transferred into harvesting solutions (2% PEG2000 MME, 40% PEG400 and 50 mM HEPES pH 7.6, 3%). The crystals were then flash-frozen in liquid nitrogen for storage and data collection under cryogenic conditions (100K). Data were collected at the Advanced Photon Source beamline 21-ID-D. A 2.7-Å dataset of TRF1_TRFH_-PinX1_TBM_ was collected and processed by HKL2000. The complex structure was solved by molecular replacement in PHASER by using the previously published unliganded TRF1_TRFH_ structure (PDB: 1H6O) as the search model. The model was then refined using Phenix, together with manual building in Coot. In the final Ramachandran plot, the favored and allowed residues are 96.6% and 100.0%, respectively. All the crystal structural figures were generated using PyMOL.

### Co-IP (Co-Immunoprecipitation)

The TRF1 gene (amplified by primers, Table S3, No. 21, 22) was inserted into a modified pcDNA3.1 expression vector containing a 3×Flag tag at the N-terminus. It was then transfected into HeLa cells which express different constructions of PinX1. After resuspending the cells in Co-IP buffer (50 mM Tris-HCl pH 8.0, 150 mM NaCl, 1 mM EDTA, 1% NP-40, Protease Inhibitor Cocktail), the cells were lysed using ultrasonic treatment. The total protein was quantified and equalized using the BCA (Bicinchoninic Acid) Protein Assay Kits (Thermo Scientific, Cat. 23227). Strep magnetic beads (Beaver, Cat. 70808) were added to the lysates. The mixture was incubated overnight at 4 ℃, followed by washing of the magnetic beads using Co-IP buffer. Finally, the elution was performed with 2.5 mM desthiobiotin (MCE, Cat. HY-128699) in Co-IP buffer, and applied for Western blot analysis.

### ChIP-qPCR (Chromatin Immunoprecipitation-quantitative PCR)

Approximately 10^6^ cells were fixed with 1% formaldehyde at room temperature for 10 min, and terminated with 0.125 M glycine. The cells were washed with PBS, and collected via scraping and centrifugation. The cell pellet was incubated with ChIP Buffer 1 (10 mM Tris pH 7.5, 10 mM NaCl, 3 mM MgCl_2_, 0.3% NP-40, 10% glycerol, Protease Inhibitor Cocktail), and ChIP Buffer 2 (50 mM HEPES pH 7.5, 150 mM NaCl, 1% Triton X-100, 0.1% sodium deoxycholic acid, 0.1% SDS, Protease Inhibitor Cocktail) sequentially for 10 min on ice. The nuclear lysate was ultrasonically sheared (Covaris S220), and after centrifugation, the supernatant was collected, quantified. The anti-Strep antibody (25 μg of DNA corresponds to 5 μg antibody, GenScript, Cat. A01732) was added and incubated at 4°C overnight. Then protein A/G beads (5 μg antibody corresponds to 125 μg beads, MCE, Cat. HY-K0202) and ChIP dilution buffer (20 mM Tris-HCl, 2 mM EDTA, 150 mM NaCl, 0.01% SDS, 1% TritonX-100) were added into the mixture, and incubated at 4°C for 4 h. The beads were then washed sequentially with low salt immune complex buffer (50 mM Tris-HCl pH 7.5, 150 mM NaCl, 0.1% SDS, 1% Triton X-100, 2 mM EDTA), high salt immune complex wash buffer (50mM Tris-HCl pH 7.5, 500mM NaCl, 2mM EDTA, 0.1% SDS, 1% Triton X-100), immune complex wash buffer (10 mM Tris-HCl pH 7.5, 0.25 M LiCl, 1 mM EDTA, 1% Igepal-CA630, 1% sodium deoxycholate), and TE buffer (10 mM Tris-HCl pH 8, 10 mM EDTA). The DNA was eluted by fresh elution buffer (1% SDS, 0.1 M NaHCO_3_), and digested in the presence of RNase A at 65°C overnight. This was followed by addition of Protease K and incubation at 55°C for 1 h. Finally, the DNA was purified by Zymo ChIP DNA Clean Concentrator kit (Integratedsci, Cat. D5201) according to the manufacturer’s instruction. Following purification, the DNA was subjected to qPCR via Roche’s Light Cycle 480 system. The analysis was performed with Telc, Telg primers (Table S3, No. 25, 26) and Hieff^®^ qPCR SYBR Green Master Mix (Yeasen, Cat. 11201ES03).

### Telomerase reconstitution, purification & pull-down assay

To express TERT and TERC, pcDNA3.1-3×Flag-TERT (amplified by primers, Table S3, No. 23, 24, and cloned into a modified pcDNA3.1) and pcDNA3.1-U3-TERC-1-451-HDV were transfected into Tc6-3 cells with PEI (polyethylenimine, Polysciences, Cat. 24765)^3^. After 60 h, the cells were harvested and resuspended in buffer T containing 20 mM HEPES, pH 7.9, 200 mM KCl, 10% glycerol, 2 mM MgCl_2_, 1 mM EDTA, 0.1% IgePal CA-630, 1 mM PMSF, 0.1 mg/ml lysozyme, 2 mM β-mercaptoethanol, Protease Inhibitor Cocktail, RiboLock RNase Inhibitor (Applied Biosystems, Cat. N8080119) before cryomilling. The cell debris was removed by ultracentrifugation, while the supernatant was purified by anti-Flag resins (GenScript, Cat. L00425). Purified telomerase was then mixed with GST-PinX1 and GST beads overnight at 4 ℃. It was then washed with buffer T and eluted with 15 mM L-Glutathione Reduced followed by Western blot analysis.

### Isolation of RNA and RT-qPCR (reverse transcription-qPCR) Analysis

RNA was isolated using the TRIzol Reagent (Invitrogen, Cat. 15596026) according to the manufacturer’s instructions. Subsequently, the RNA sample was then reverse-transcribed into cDNA with Hifair^®^ Ⅲ Reverse Transcriptase (Yeasen, Cat. 11141ES60). After reverse-transcription, the cDNA was subjected to qPCR analysis with Hieff^®^ qPCR SYBR Green Master Mix and the corresponding primers (Table S3, No. 27-32) using Roche’s Light Cycle 480 system. For PinX1 quantification, GAPDH was used as an endogenous control. Quantification of the relative levels was determined by the comparative threshold cycle method.

### Western blot analysis

Samples (cell lysates, products of Co-IP or pull-down assays) were subjected to SDS-PAGE separation and then blotted onto PVDF membranes (Millipore). The blots were incubated in blocking buffer (5% fat-free milk in PBS buffer supplemented with 0.05% TWEEN-20) at room temperature for 1 h, and incubated with primary antibodies in blocking buffer at 4°C for overnight (PinX1, 1:1000, ABclonal, Cat. A17172; GAPDH, 1:8000, Proteintech, Cat. 60004; GST, 1:5000, Proteintech, Cat. 66001; Flag, 1:5000, ABclonal, Cat. AE063; Strep, 0.25 μg/mL, GenScript, Cat. A01732). Blots were washed and incubated with HRP-labeled secondary antibodies at room temperature for 1 h. After washing, blots were developed with ECL Prime Western Blotting System (GE Healthcare, Cat. RPN2232).

### TRAP (telomeric repeat amplification protocol) assay

TRAP assay was performed as described previously^6^. The sample (cell lysates or purified proteins) was incubated in a specific reaction system consisting of 20 mM Tris-HCl pH 8.3, 1.5 mM MgCl_2_, 63 mM KCl, 0.05% Tween 20, 1 mM EGTA, 50 μM dNTP, 0.2 μM TS primer (Table S3, No. 33), 0.2 μM ACX primer (Table S3, No. 34), 0.2 μM NT primer (Table S3, No. 35), 2×10^-16^ μM TSNT primer (Table S3, No. 36), 0.4 mg/mL BSA, and 0.04 U/μL Taq DNA polymerase at 25 ℃ for 40 min for extension, before being amplified by PCR. The PCR products were then resolved on a 10% polyacrylamide gel and silver stained. The telomerase activity was quantified by analyzing the bands gradient using ImageJ software (2.3.0).

### Q-FISH (quantitative Fluorescence in Situ Hybridization) telomere length analysis

Once the cell density in the 10 cm dish reached 50%, the colchicine (MCE, Cat. HY-N0282) was added at a final concentration of 1 μg/mL. Following a 1-h incubation, the cells were digested and harvested. The cells were then treated with pre-warmed 50 mM KCl and incubated at 37 ℃ for 30 min. After centrifugation, the supernatant was then removed, and the cells were gently washed drop-by-drop with the pre-cooled fixing solution (methanol: glacial acetic acid = 3:1). The cells were stored overnight in the fixing solution at 4 ℃. Then the cells were suspended in an appropriate amount of fresh fixative (~5 million/mL), and dropped onto a slide at an appropriate height. The slide was then placed on a wet tissue at 80 ℃ for approximately 1 minute to vaporize the fixative by steam, and left to dry for a minimum of 2 h. Subsequently, the slide was soaked in PBS at room temperature, and fixed with 4% formaldehyde-PBS. The slide was then washed with PBS, and digested with a 1 mg/mL pepsin solution at 37 ℃ for 5 min, followed by another wash with PBS and a soak in PBS at room temperature. Following this, the slide was fixed and rewashed. Ethanol gradient dehydration was performed, followed by drying. Next, the PNA probe (TelC-Cy3, TAHE, Cat. F1002, CCCTAACCCTAACCCTAA) was diluted with the hybrid solution (50 nM in 10 mM Tris-HCl pH 7.2, 70% formamide, 0.5% sealing solution, Roche, Cat. 11096176001) and preheated. The slide was hybridized with the probe and placed at 80 ℃ for 10 min. Then the slide was incubated at room temperature overnight, being shielded from light and kept moist. Following hybridization, the slide was washed twice with Wash-1 (10 mM Tris-HCl pH 7.2, 0.1% BSA, and 70% formamide). Subsequently, the slide was washed three times with Wash-2 (0.1M Tris-HCl pH 7.2, 150mM NaCl, and 0.08% Tween-20), followed by DAPI (Yeasen, Cat. 40728ES03) dyeing. The slide was then dehydrated using an ethanol gradient, and allowed to dry. Fluorescent images were captured by Leica Lightning using 63× oil-immersion objective. Values of the telomere fluorescence were measured utilizing Imaris software (9.3.1).

### Statistical Analysis

All statistical analyses were performed using R (4.2.0) or GraphPad software and were described in the figure legends. Correlations were reported as Spearman’s rank correlation coefficient (Fig. S1A; Fig. S6D, E). For survival analysis, hazard ratios were estimated with Cox proportional hazards model, and p values were calculated by log-rank test (Fig. S1F-L). For comparison of two independent groups, the Student’s t-test (Fig. 1A, B; Fig. S2D-P; Fig. S5D) or Wilcoxon rank sum test (Fig. S1B-E) was employed. For comparison of three or more independent groups, the t-test was adjusted by false discovery rate (Fig. 1E, M; Fig. S2A, B; Fig. S4G; Fig. S6F, G; Fig. S7A-I). All statistical significances were determined with two-tailed tests. The data (Fig. 1A, B, E, I, M; Fig. S2A, B, D-P; Fig. S4G; Fig. S5D; Fig. S6F, G; Fig. S7A-I) were represented as mean ± standard deviation from three independent experiments.

## Reference

1. Barthel FP, Wei W, Tang M, et al. Systematic analysis of telomere length and somatic alterations in 31 cancer types. *Nat Genet*. 2017;49(3):349-357. doi:10.1038/ng.3781

2. Noureen N, Wu S, Lv Y, et al. Integrated analysis of telomerase enzymatic activity unravels an association with cancer stemness and proliferation. *Nat Commun*. 2021;12(1):139. doi:10.1038/s41467-020-20474-9

3. Wan F, Ding Y, Zhang Y, et al. Zipper head mechanism of telomere synthesis by human telomerase. *Cell Res*. Published online November 15, 2021. doi:10.1038/s41422-021-00586-7

4. Borowicz S, Van Scoyk M, Avasarala S, et al. The soft agar colony formation assay. *J Vis Exp*. 2014;(92):e51998. doi:10.3791/51998

5. Bahmad HF, Cheaito K, Chalhoub RM, et al. Sphere-Formation Assay: Three-Dimensional in vitro Culturing of Prostate Cancer Stem/Progenitor Sphere-Forming Cells. *Front Oncol*. 2018;8:347. doi:10.3389/fonc.2018.00347

6. Herbert BS, Hochreiter AE, Wright WE, Shay JW. Nonradioactive detection of telomerase activity using the telomeric repeat amplification protocol. *Nat Protoc*. 2006;1(3):1583-1590. doi:10.1038/nprot.2006.239
