# Supplementary material for: PinX1 suppresses cancer progression by inhibiting telomerase activity in cervical squamous cell carcinoma and endocervical adenocarcinoma
Source: Genes Dis. 2024 May 7;12(2):101319. doi: 10.1016/j.gendis.2024.101319 (PMC11615109; doi:10.1016/j.gendis.2024.101319)
Supplement: Multimedia component 2 [file mmc2.docx]

### Figure S1 PinX1 deletion correlates with poor prognosis in the TCGA dataset. (A) Scatterplot of multiple TCGA projects ranking by telomere length and telomerase activity. Tumor types with fewer than 10 samples were excluded. CESC (cervical squamous cell carcinoma and endocervical adenocarcinoma) was highlighted in red. The R and *P* values were calculated by the Spearman rank correlation test. (B) Comparison of PinX1 mRNA levels in different PinX1 gene states in CESC. The *P* value was calculated with the Wilcoxon rank sum test. (C) Comparison of telomerase activity between different PinX1 gene states in CESC. The *P* value was calculated with the Wilcoxon rank sum test. (D, E) Comparison of cancer stemness between different PinX1 gene states in CESC and pan-cancer. The *P* values were calculated with the Wilcoxon rank sum tests. (F, G) Kaplan-Meier overall survival and disease-specific survival curves for patients with or without PinX1 deletion in CESC. HRs (hazard ratios) were estimated with the Cox models, and *P* values were calculated with the log-rank tests. (H, I) Kaplan-Meier overall survival and disease-specific survival curves for patients with or without PinX1 deletion in pan-cancer. HRs were estimated with the Cox models, and *P* values were calculated with the log-rank tests. (J–L) Overall survival of patients with or without PinX1 deletion in UCEC (uterine corpus endometrial carcinoma), KIRP (kidney renal papillary cell carcinoma), and UVM (uveal melanoma). HRs were estimated with the Cox models, and *P* values were estimated with the log-rank tests.

### Figure S2 PinX1 deletion correlates with cancer aggressiveness. (A) shRNA screening for PinX1 knockdown in HeLa cells. Knockdown efficiency was examined by real-time quantitative PCR. The *t*-tests adjusted by false discovery rate were used for statistical analysis; ^***^*P* < 0.001. (B) Growth curves of HeLa cells stably transfected with shCtrl, shPinX1-1, shPinX1-2, or shPinX1-3 were recorded using the RTCA. The *t*-tests adjusted by false discovery rate were used to analyze the differences between shCtrl and shPinX1-1 at 36, 48, 60, 72, 84, and 96 h respectively; ^**^*P* < 0.01. shPinX1-1 was selected for further experiments, termed as shPinX1 in this study. (C) Knockdown of PinX1 in HeLa, CaSki, and SiHa cells was confirmed by western blot. (D, E) Growth curves of shCtrl and shPinX1 CaSki/SiHa cells were recorded using the RTCA. The *P* values were calculated with the student's *t*-tests. (F, G) Quantification of the colony formation of shCtrl and shPinX1 CaSki/SiHa cells in soft agar. Representative images for crystal violet staining and colonies were shown. The *P* values were calculated with the student's *t*-tests. Scale bar, 100 μm. (H–M) Quantification of the migration and invasion of shCtrl and shPinX1 HeLa/CaSki/SiHa cells. Representative crystal violet staining images were shown. The *P* values were calculated with the student's *t*-tests. Scale bar, 100 μm. (N–P) Dose-response curves of shCtrl and shPinX1 HeLa/CaSki/SiHa cells. The IC50s (50% inhibitory concentrations) of cisplatin were labeled on the graph. The student's *t*-tests were used to analyze the differences at certain concentrations; ^*^*P* < 0.05, ^**^*P* < 0.01, ^***^*P* < 0.001. CI, confidence interval.

### Figure S3 Structure of the TRF1_TRFH_-PinX1_TBM_ complex. (A) The gel filtration chromatography (Superdex 200) and SDS-PAGE gel verification of the purified TRF1_TRFH_-PinX1_TBM_ complex. (B) The overall structure of the dimeric TRF1_TRFH_-PinX1_TBM_ complex. (C) Electron density map of PinX1_TBM_.

### Figure S4 Telomerase inhibition by GST-PinX1 C-terminal truncations and mutants. (A) The impact of PinX1 truncations on telomerase activity was analyzed by TRAP assays. "- ~ ++++" indicated an increasing gradient of RTI (relative telomerase inhibition) (the same as in panel B–D, F, G). (B) TRAP assays of RTI for different GST-PinX1 C-terminal truncations. (C) Quantification of panels A, B. (D) TRAP assays of RTI for different GST-PinX1_132-328_ mutants. (E) Quantification of TRAP signals from different GST-PinX1 C-terminal truncations. CTD (C-terminal domain), GST-PinX1_132-328_. (F) The impact of GST-PinX1_132-328_ and GST-PinX1_132-328 (MUT)_ on telomerase activity was analyzed by TRAP assays. (G) Quantification of panel F. The experiment was conducted three times and statistically analyzed by the *t*-tests adjusted by false discovery rate; ^***^*P* < 0.001. RTA, relative telomerase activity.

### Figure S5 TID (telomerase inhibitory domain)-mediated direct binding and inhibition of PinX1 on purified telomerase. (A) Human telomerase was purified, and verified by SDS-PAGE silver staining and western blot. (B) SDS-PAGE silver staining for pull-down assays of 3×Flag-TERT captured by GST-PinX1_132-328_. (C) Western blot verification for pull-down assays of 3×Flag-TERT captured by GST and GST-PinX1_132-328_. (D) Real-time quantitative PCR of TERC (telomerase RNA component) pulled down by GST and GST-PinX1_132-328_. The *P* value was calculated with the student's *t*-test. (E) The impact of GST-PinX1 C-terminal truncations on purified telomerase was analyzed by TRAP assays. IC, internal control. (F) The capacity of variant PinX1 truncations for binding to and inhibiting telomerase. The increasing binding capacity was indicated by "- ~ +++". The increasing gradient of RTI was indicated by "- ~ ++++".

### Figure S6 PinX1 suppresses cancer stemness through the inhibition of telomerase activity. (A) 2×Strep tagged-PinX1_2-328_ (WT), -PinX1_203-328_ (TID), and -PinX1_2-328 (MUT292-301)_ (MUT) were expressed in HeLa, CaSki, and SiHa cells, and confirmed by western blot. (B, C) Quantitative fluorescence *in situ* hybridization analysis and statistics of telomere length in CaSki and SiHa cells expressing different PinX1 constructions. Scale bar, 10 μm. MTF, mean telomere fluorescence. (D, E) Correlation analysis of telomerase activity and cancer stemness in CESC and pan-cancer. The R and *P* values were calculated by the Spearman rank correlation test. (F, G) Representative images and statistics of sphere formation of CaSki and SiHa cells expressing different PinX1 constructions. Scale bar, 100 μm.

### Figure S7 Suppression of PinX1 on cancer aggressiveness. (A–C) Crystal violet staining and statistics of migration and invasion in HeLa, CaSki, and SiHa cells expressing different PinX1 constructions. Scale bar, 100 μm. (D–F) HeLa, CaSki, and SiHa cells expressing different PinX1 constructions were treated with cisplatin (HeLa: 7.5 μg/mL; CaSki and SiHa: 1 μg/mL) for 3 days. Cell viability was measured by SRB assay. (G–I) Growth curves of HeLa, CaSki, and SiHa cells expressing different PinX1 constructions were recorded using the RTCA. All statistics were performed using the *t*-tests adjusted by false discovery rate; n.s., *P* > 0.05; ^*^*P* < 0.05, ^**^*P* < 0.01, ^***^*P* < 0.001.

### Figure S8 Schematic diagram of a tumor suppressive effect of PinX1 in cervical squamous cell carcinoma and endocervical adenocarcinoma (CESC) by telomerase inhibition. We propose that PinX1 is recruited to telomeres by TRF1, and subsequently binds to telomerase and inhibits its activity, resulting in a tumor-suppressive effect on CESC (top). In cancers, PinX1 deletion unleashes telomerase activity to elongate telomeres and contributes to tumor progression (bottom).
