# Supplementary figures and images for: PinX1 suppresses cancer progression by inhibiting telomerase activity in cervical squamous cell carcinoma and endocervical adenocarcinoma
Source: Genes Dis. 2024 May 7;12(2):101319. doi: 10.1016/j.gendis.2024.101319 (PMC11615109; doi:10.1016/j.gendis.2024.101319)

Table S2. Crystal data collection and refinement statistics


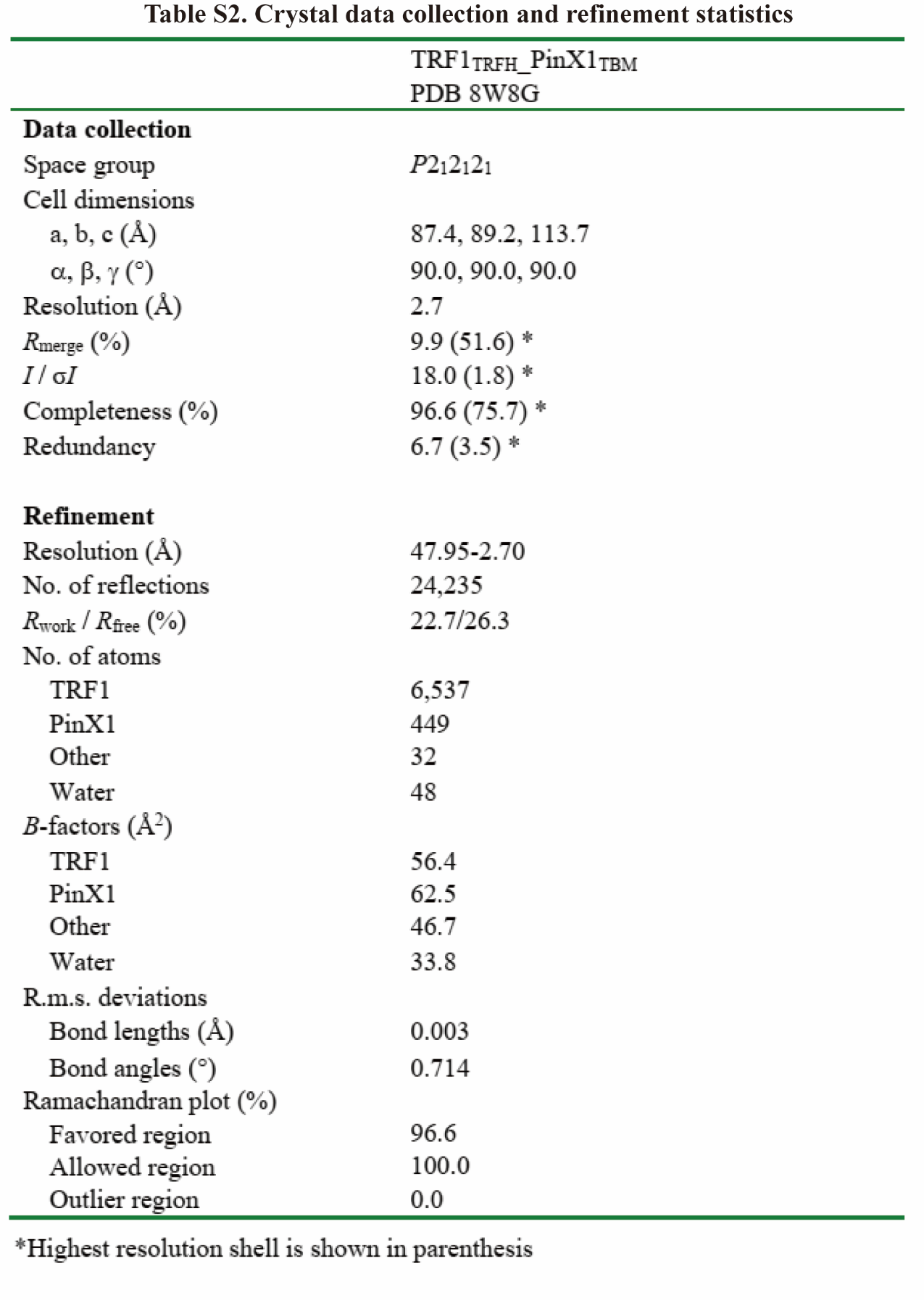

Supplement: Multimedia component 4 [file mmc4.docx]
